# Novel Approach in Cardiac Sarcoidosis: A Case Report Highlighting Abatacept as a Promising Treatment Option

**DOI:** 10.7759/cureus.45805

**Published:** 2023-09-23

**Authors:** Maamannan Venkataraj, Tyler Pierotti, Manush Sondhi, Shravya Balmuri, Samina Hayat

**Affiliations:** 1 Internal Medicine, Louisiana State University Health Sciences Center, Shreveport, USA; 2 Rheumatology, Louisiana State University Health Sciences Center, Shreveport, USA

**Keywords:** cardiology, rheumatology, abatercept, sarcoidosis, cardiac sarcoidosis

## Abstract

Cardiac sarcoidosis (CS) is a rare auto-immune disorder where immune cells form granulomas in the heart that may lead to potential arrhythmias and heart failure. Due to the low prevalence of CS, the management remains challenging, requiring a multidisciplinary approach. In addition to the management of the resulting arrhythmias and heart failure, corticosteroids and immunosuppressants are used as anti-inflammatories to prevent disease progression. Immunosuppressive regimens for the treatment of CS are not yet well established. Abatacept has been approved for rheumatoid arthritis and psoriatic arthritis and both are mainly Th1-driven autoimmune diseases. Even though there are several different drugs used to treat corticosteroid-dependent sarcoidosis, abatacept may represent a unique option as its side effects differ from other drugs like methotrexate, azathioprine, or mycophenolate, especially bone marrow and liver toxicity. We present the case of a 52-year-old CS patient treated with abatacept after the failure of methotrexate and mycophenolate mofetil. Our patient had a history of stage D heart failure with reduced ejection fraction (HFrEF) with ejection fraction (EF) of 15-20%, nonischemic cardiomyopathy (NICM) s/p left heart catheterization (LHC), CS diagnosed by positron emission tomography (PET), status post implantable cardioverter-defibrillator (ICD) implantation, lung sarcoid, paroxysmal atrial fibrillation, and aflutter, who followed with cardiology, rheumatology, and pulmonology. He had multiple admissions for heart failure exacerbations. The patient was initially diagnosed with pulmonary sarcoidosis after which he completed a small course of steroids. CT chest showed lymphadenopathy; however, endobronchial ultrasound (EBUS) did not show evidence of pulmonary sarcoidosis. During an admission for heart failure about four years later, cardiac PET CT showed CS, and rheumatology was brought on board. The patient initially refused steroids and steroid-sparing agents. At subsequent visits, the patient was amenable to medication and was started on methotrexate 10mg weekly. However, given worsening chronic kidney disorder, methotrexate was discontinued and mycophenolate 200mg daily was started. A couple of weeks after mycophenolate was started, the patient felt like “his throat was closing up” and his stomach was cramping, which was thought to be an allergic response to the mycophenolate so it was discontinued. He then received an abatacept infusion which he tolerated well. Currently, our patient has been referred for heart transplantation.

## Introduction

Sarcoidosis is a systemic disorder characterized by noncaseating granulomatous inflammation in multiple organs. While the lungs are primarily affected, sarcoidosis can involve other organs as well. Cardiac sarcoidosis (CS) is a rare autoimmune condition associated with a worse prognosis, characterized by the formation of granulomas in the heart. CS can lead to restrictive cardiomyopathy, pericardial effusion, atrioventricular block, and even sudden cardiac death. Diagnosing CS is challenging due to its patchy involvement, resulting in low sensitivity of endomyocardial biopsy. Imaging modalities play a crucial role in evaluating and managing patients with CS. Given the low prevalence of CS, comprising 2-5% of patients with systemic sarcoidosis, its management requires a multidisciplinary approach. In addition to managing arrhythmias and heart failure, anti-inflammatory agents such as corticosteroids and immunosuppressants are used to prevent disease progression. However, optimal immunosuppressive regimens for CS remain uncertain. This report presents an intriguing case of CS diagnosed using a positron emission tomography (PET) scan, unable to tolerate methotrexate and mycophenolate mofetil, and successfully treated with abatacept. This case highlights the challenges of managing CS and the potential therapeutic role of abatacept in refractory patients.

## Case presentation

A 52-year-old African American male with a past medical history of pulmonary sarcoidosis, non-ischemic cardiomyopathy (NICM) status post-left heart catheterization in 2015, heart failure with reduced ejection fraction (EF) with EF of 15-20%, status post implantable cardioverter-defibrillator (ICD) implantation in 2018, paroxysmal atrial fibrillation, and aflutter presented to our facility with dyspnea. The patient had a history of multiple admissions for heart failure exacerbations since his initial diagnosis of NICM. He received follow-up care from our institution's cardiology, rheumatology, and pulmonology services. Prior to his presentation to our facility, the patient had initially sought medical attention at another hospital in 2015, where he was being evaluated for presumed sarcoidosis and an unknown cardiomyopathy. During this period, the patient received treatment with steroids for an unspecified duration. 

**Figure 1:**
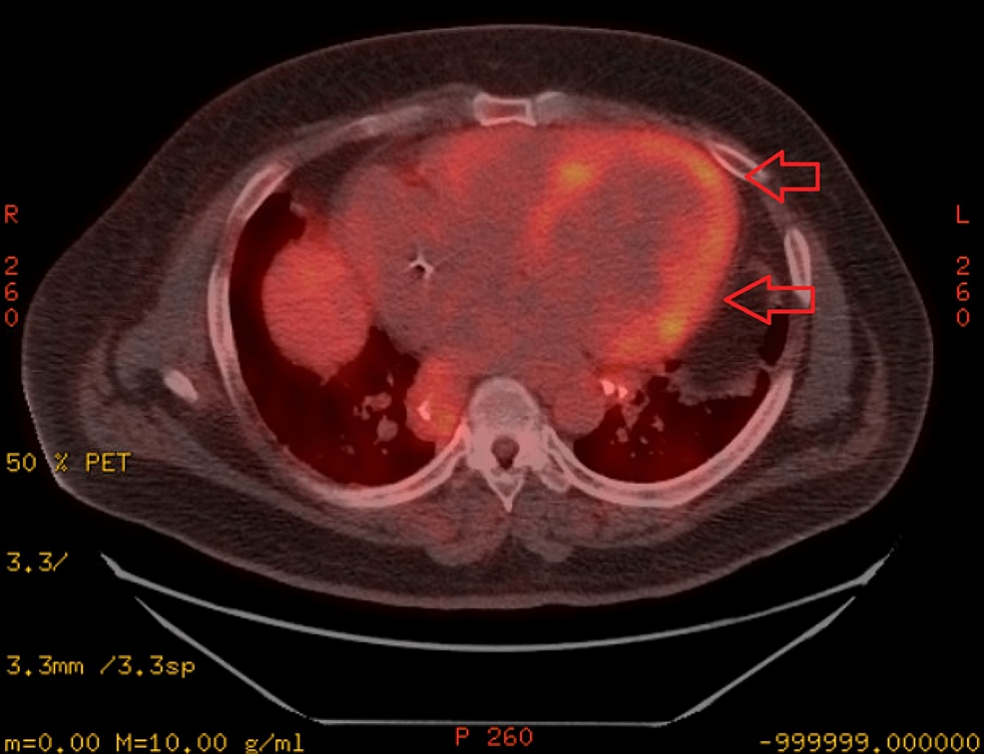
PET scan axial view revealing diffuse FDG uptake, most prominent in the anterior, anterolateral, and inferior wall, consistent with diffuse active sarcoidosis PET: positron emission tomography; FDG: fluorodeoxyglucose

**Figure 2:**
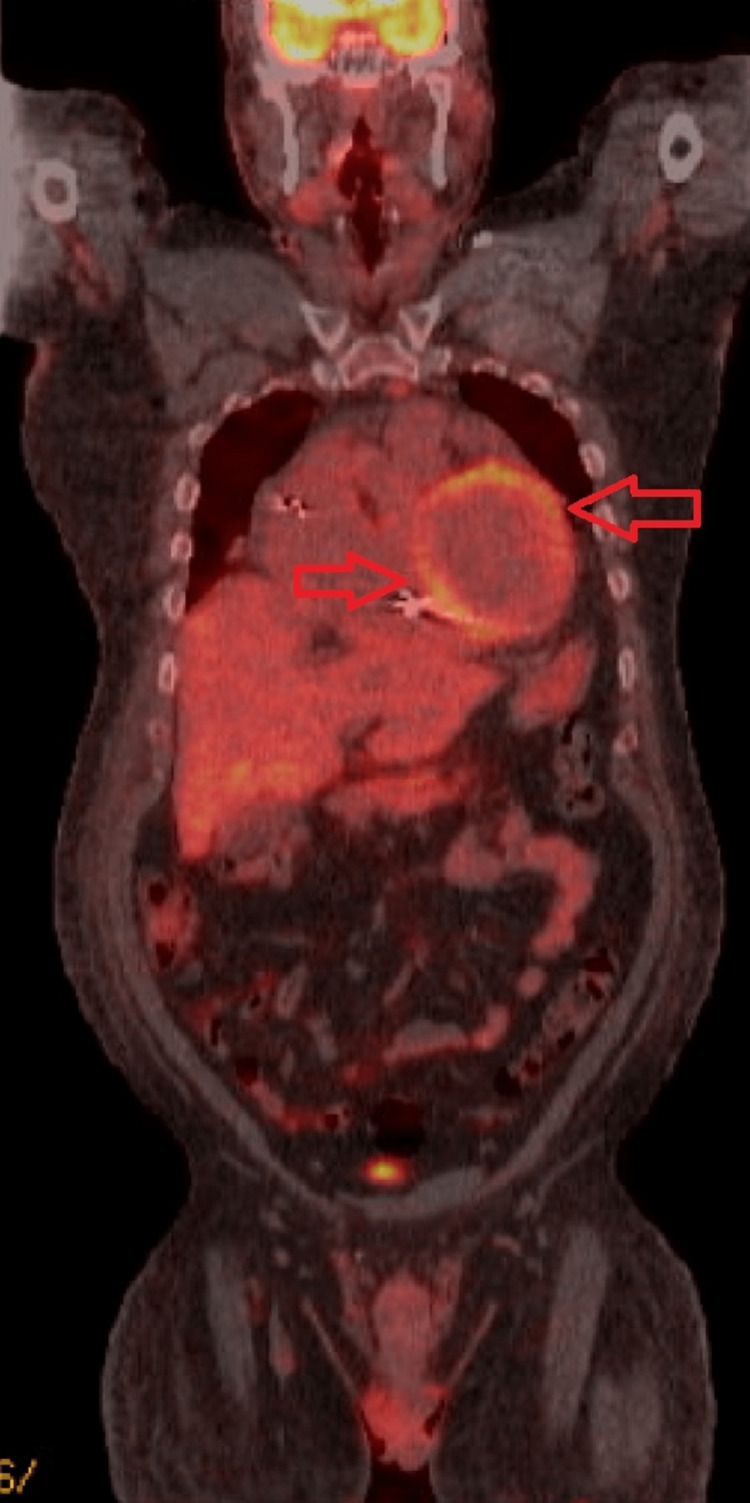
PET scan coronal view revealing diffuse FDG uptake in the heart PET: positron emission tomography; FDG: fluorodeoxyglucose

The patient presented to our facility with worsening dyspnea. A chest computed tomography (CT) scan revealed bilateral ground glass opacities and mediastinal lymphadenopathy, raising suspicion of pulmonary sarcoidosis. However, endobronchial ultrasound (EBUS) did not provide evidence of pulmonary sarcoidosis. Further evaluation during admission for heart failure exacerbation involved a cardiac positron emission tomography (PET)-CT scan, which demonstrated diffuse fluorodeoxyglucose (FDG) uptake. The FDG uptake was most prominent in the anterior, anterolateral, and inferior walls from the base to distal regions and in the anterolateral papillary muscle as seen in Figures 1-2. These findings were consistent with diffuse active sarcoidosis involving the heart.

Initially, the patient was reluctant to initiate treatment with steroids or steroid-sparing agents. However, during subsequent visits, he expressed a willingness to pursue medication management. As a result, methotrexate was initiated at a dose of 10mg weekly. Unfortunately, the patient experienced a decline in kidney function after initiating methotrexate, leading to the discontinuation of this medication due to worsening chronic kidney disease (CKD). Subsequently, the patient was started on mycophenolate mofetil at a dose of 200mg daily. However, after a couple of weeks, the patient developed an allergic reaction characterized by throat tightness and abdominal cramping. As a result, mycophenolate mofetil was discontinued.

Given the extensive cardiac involvement with a reduced EF, the patient was started on abatacept in September 2022. Abatacept, an immunomodulator that interferes with T-cell activity, was chosen over azathioprine due to its potential efficacy in controlling ongoing inflammation as well as its noted cardioprotective effects when compared to tumor necrosis factor-alpha (TNF-α) inhibitors. The patient tolerated abatacept well, and subsequent follow-up assessments revealed no further drop in his EF. The patient has been referred for heart transplantation due to the advanced nature of his CS and heart failure.

## Discussion

CS is characterized by the infiltration of epithelioid cell granulomas into the myocardium, leading to various cardiac complications such as conduction disturbances, ventricular tachyarrhythmias, and heart failure. However, it is essential to recognize that manifest CS represents only the visible tip of the iceberg. Advanced imaging techniques have revealed that cardiac involvement occurs far more frequently, approximately four to five times, than what is clinically evident [1]. This hidden aspect of CS underscores the need for heightened vigilance and comprehensive evaluation to detect and manage the condition effectively. The broader prevalence of cardiac involvement highlights the importance of early diagnosis and targeted interventions to prevent potential complications and optimize patient outcomes. Timely detection and a comprehensive approach are crucial for preventing life-threatening arrhythmias and sudden cardiac death. While the management of CS relies on an individualized treatment regimen, immunosuppressants become necessary in cases with an underlying high-grade atrioventricular (AV) block, atrial and ventricular arrhythmias, and reduced left ventricular (LV) function [2].

Corticosteroids remain the first-line therapy for active CS due to their non-selective suppression of cytokine production responsible for granuloma formation. Corticosteroids limit the movement of inflammatory cells, restore CD4+ T cell function, and rebalance the subset of effector CD4 T cells involved. The specific dosage of corticosteroids depends on the severity of the initial presentation and the extent of cardiac involvement [3].

Incorporating steroid-sparing drugs in the early stages of treatment is beneficial to achieve early remission and minimize the need for long-term steroid use. Methotrexate, a second-line agent or adjuvant to corticosteroids, has shown promising results. Studies have demonstrated a high rate of inflammation remission on FDG-PET and stable levels of LV EF and N-terminal pro-B-type natriuretic peptide (NT-proBNP) after three years of treatment with methotrexate in combination with corticosteroids [4,5].

The secretion of TNF by macrophages is pivotal in sarcoid granuloma maintenance and formation. Biologic anti-TNF agents can prevent granuloma formation and are effective in treating CS when other therapies fail. Infliximab, a chimeric TNF antibody, is well-tolerated at doses <10 mg/kg even in patients with impaired LV function. Recent systematic reviews by Adler et al. have shown that TNF-α inhibitors significantly improve vital capacity and lead to a 79% improvement in pulmonary function [6]. These inhibitors have also demonstrated efficacy in extrapulmonary sarcoidosis, making them a potential third-line therapy.

In addition to TNF-α inhibitors, abatacept, an immunomodulator that interferes with T cell activity, has shown promise in treating sarcoidosis, as it has been successfully used in managing rheumatoid arthritis and psoriatic arthritis. In a multicenter phase II study by Frye et al. in 2020, abatacept, combined with corticosteroids, was administered to 30 chronic sarcoidosis patients for one year. The study hypothesized that abatacept effectively controls ongoing inflammation and can be safely administered in patients with sarcoidosis [7].

Notably, Kang et al. conducted a cohort study in 2018 involving a large number of patients with underlying rheumatoid arthritis and found that abatacept was associated with a reduced number of adverse cardiovascular outcomes. The study revealed no significant difference in heart failure risk compared to other TNF-α inhibitors in biologic-naïve patients [8]. Similarly, Jin et al., in their observational studies published in 2018, analyzed primary and secondary outcomes in rheumatoid arthritis patients with and without baseline cardiovascular disease. Their findings indicated a protective trend with abatacept compared to TNF-α inhibitors [9].

Considering our patient's persistent symptoms, we initiated abatacept therapy following a trial of steroids, methotrexate, and mycophenolate mofetil. This decision was based on the protective trend and better vascular outcomes associated with abatacept compared to TNF-α inhibitors as seen in the study by Jin et al. [9], as well as the hypothesis that abatacept can be used for therapy in CS noted by Frye et al. [7]. Since initiating abatacept treatment, our patient's EF remained stable and he has had a reduced number of hospitalizations for heart failure.

## Conclusions

At present, the treatment approach for CS entails corticosteroid-based immunosuppression to manage myocardial inflammation, coupled with medical and device-based therapies to address symptomatic atrioventricular block, ventricular tachyarrhythmias, and heart failure. Recent outcome data reveals promising five-year survival rates of 90-96% for manifest CS, with 10-year figures ranging from 80% to 90%. However, substantial advancements in CS care hinge on unlocking the molecular-genetic pathogenesis and conducting large-scale controlled clinical trials to further enhance treatment strategies and patient outcomes.
